# Geochemical and sedimentary constraints on the formation of the Venta Micena early Pleistocene site (Guadix-Baza Basin, Spain)

**DOI:** 10.1038/s41598-021-01711-7

**Published:** 2021-11-17

**Authors:** Alejandro Granados, Oriol Oms, Pere Anadón, Jordi Ibáñez-Insa, Anu Kaakinen, Juan Manuel Jiménez-Arenas

**Affiliations:** 1 Departamento de Ecología y Geología, Universidad de Málaga, Campus Teatinos, 29071 Málaga, Spain; 2 Fac. Ciències, Department de Geologia (Unitat d’Estratigrafia), Universitat Autònoma de Barcelona, 08193 Bellaterra, Spain; 3 Institut de Ciències de la Terra Jaume Almera (ICTJA-CSIC), Lluís Solé Sabarís s/n, 08028 Barcelona, Spain; 4 Geosciences Barcelona (GEO3BCN-CSIC), Lluís Solé Sabarís s/n, 08028 Barcelona, Spain; 5 Department of Geosciences and Geography, University of Helsinki, (Gustaf Hällströmin katu 2), P.O. Box 64, 00014 Helsinki, Finland; 6 Departamento de Prehistoria y Arqueología. Campus de Cartuja S/N, Universidad de Granada, 18071 Granada, Spain

**Keywords:** Geochemistry, Limnology

## Abstract

Despite the paleontological relevance of the terrestrial Early Pleistocene Venta Micena bonebed (Baza Basin, Spain), it lacks a comprehensive geochemical/sedimentological study. Here, we demonstrate that the 1.5-m-thick Venta Micena limestone formed in a relatively small freshwater wetland/pond located at the periphery of the large saline Baza paleolake. Two microfacies are observed, with high and low contents of invertebrate fossils, and which originated in the centre and margin of the wetland, respectively. X-ray diffraction (XRD) mineralogy and paleohydrological characterization based on ostracod and bulk-rock geochemistry (δ^13^C and δ^18^O) indicate that the limestone reflects a general lowstand of the Baza lake, permitting the differentiation of freshwater wetlands that were fed by adjacent sources. Conversely, during highstands, the Baza lake flooded the Venta Micena area and the freshwater fauna was replaced by a saline one. Bulk-rock isotopic data indicate that the lower interval C1 of the limestone (bone-rich in marginal settings) displays general negative values, while the upper interval C2 (bone free) displays less negative values. The bones of predated mammals accumulated in the marginal areas, which were flooded and buried by recurring water-table fluctuations. Lake dynamics played a critical role in bone accumulation, which was previously considered as representing a hyena den.

## Introduction

**Figure 1:**
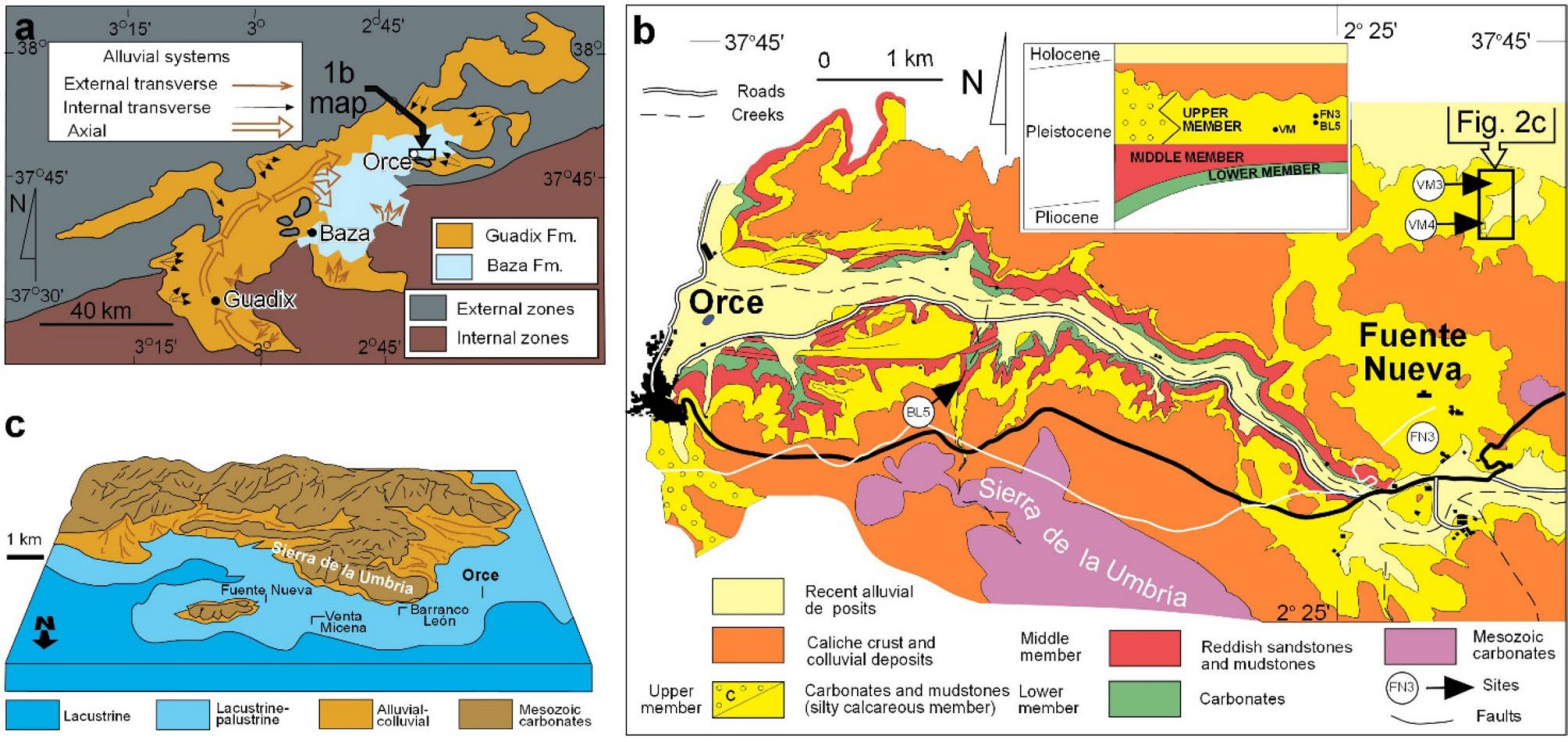
(**a**) Geological map of the Guadix-Baza basin and paleolake, with the area of the detailed map in b indicated. (**b**) Geological map^6^ of the main sites in the Orce area (BL5: Barranco León 5, FN3: Fuente Nueva 3, VM3: Venta Micena 3). The rectangle to the upper left is the area represented in Fig. 2c. (**c**) Paleogeographical setting of the Venta Micena, Barranco León and Fuente Nueva sites. Maps created with Inkscape 1.0.1 (3bc2e813f5, 2020-09-07) software (https://inkscape.org/es/).

The Venta Micena (VM) paleontological site is a reference locality for Eurasian land mammal biostratigraphy at the Early-Middle Pleistocene transition from Eurasia^1–3^. The youngest part of the Early Pleistocene interval of this transition is well dated in the paleolake succession of the Guadix-Baza basin (Fig. 1) with combined magneto-biochronology^4,5^. It encompasses the arrival of the first *Homo* genus in Europe at the nearby sites of Fuente Nueva 3 and Barranco León, the latter dated at 1.46 Ma^6,7^. The VM site provides an environmental picture of terrestrial ecosystems just before the arrival of *Homo*, despite claims that human bones existed in VM^8^.

**Figure 2:**
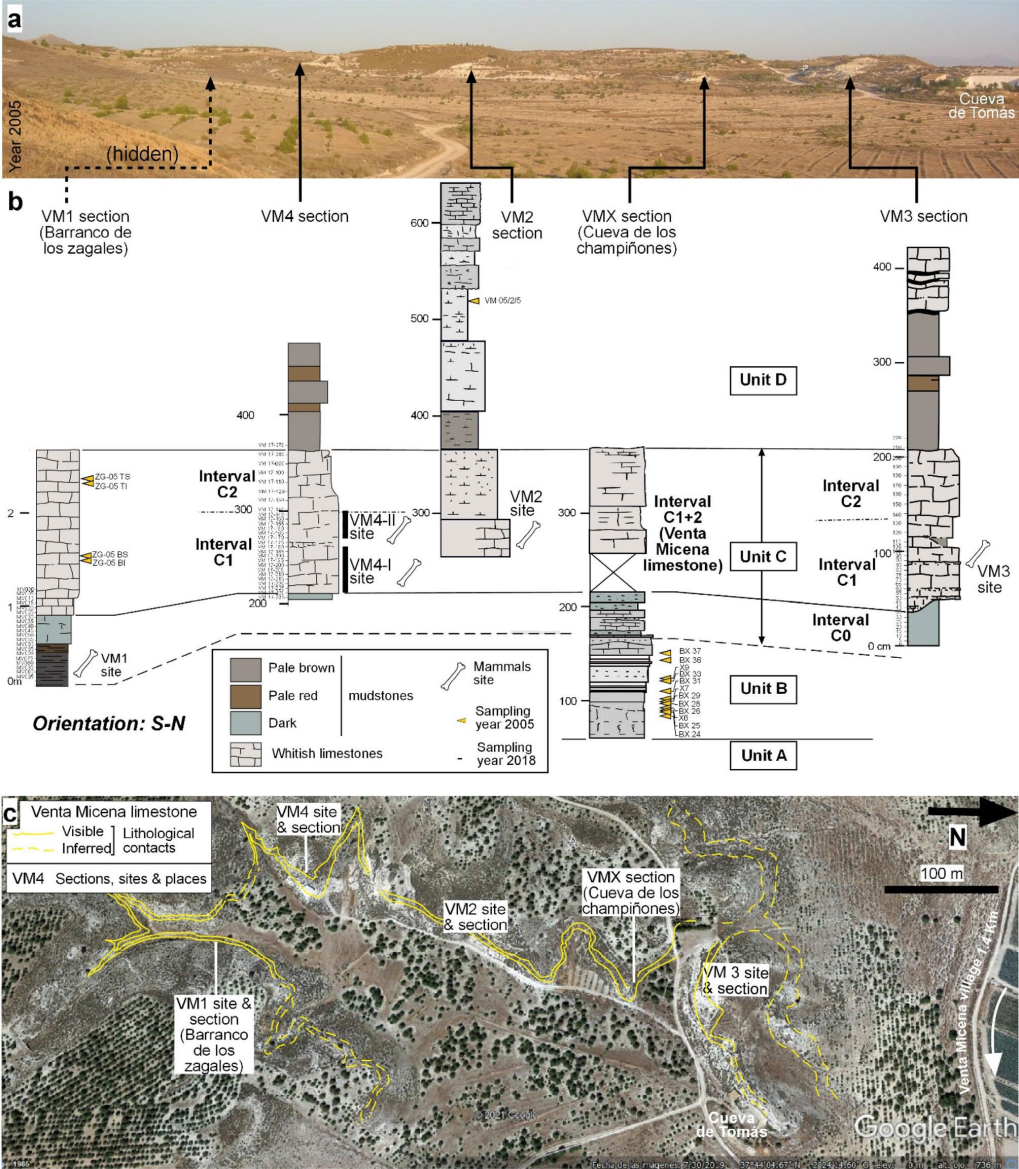
The Venta Micena (VM) limestone sections^15^ investigated in this study: (**a**) Location in a landscape ground view (own picture). (**b**) Lithology and indication of the sampling levels (see results in further figures and Supplementary Notes S1). Carbonate rocks have been differentiated on the basis of the carbonate content (figure created with Inkscape 1.0.1 software, https://inkscape.org/es/). (**c**) Satellite image (Google EARTH and Instituto Geográfico Nacional, Spain) with a rough indication of the limestone distribution. ‘Cueva de Tomás’ is the cave house that was the reference section including VM3^15^. Additional stratigraphic data and results are presented in Supplementary Notes S2.

**Figure 3:**
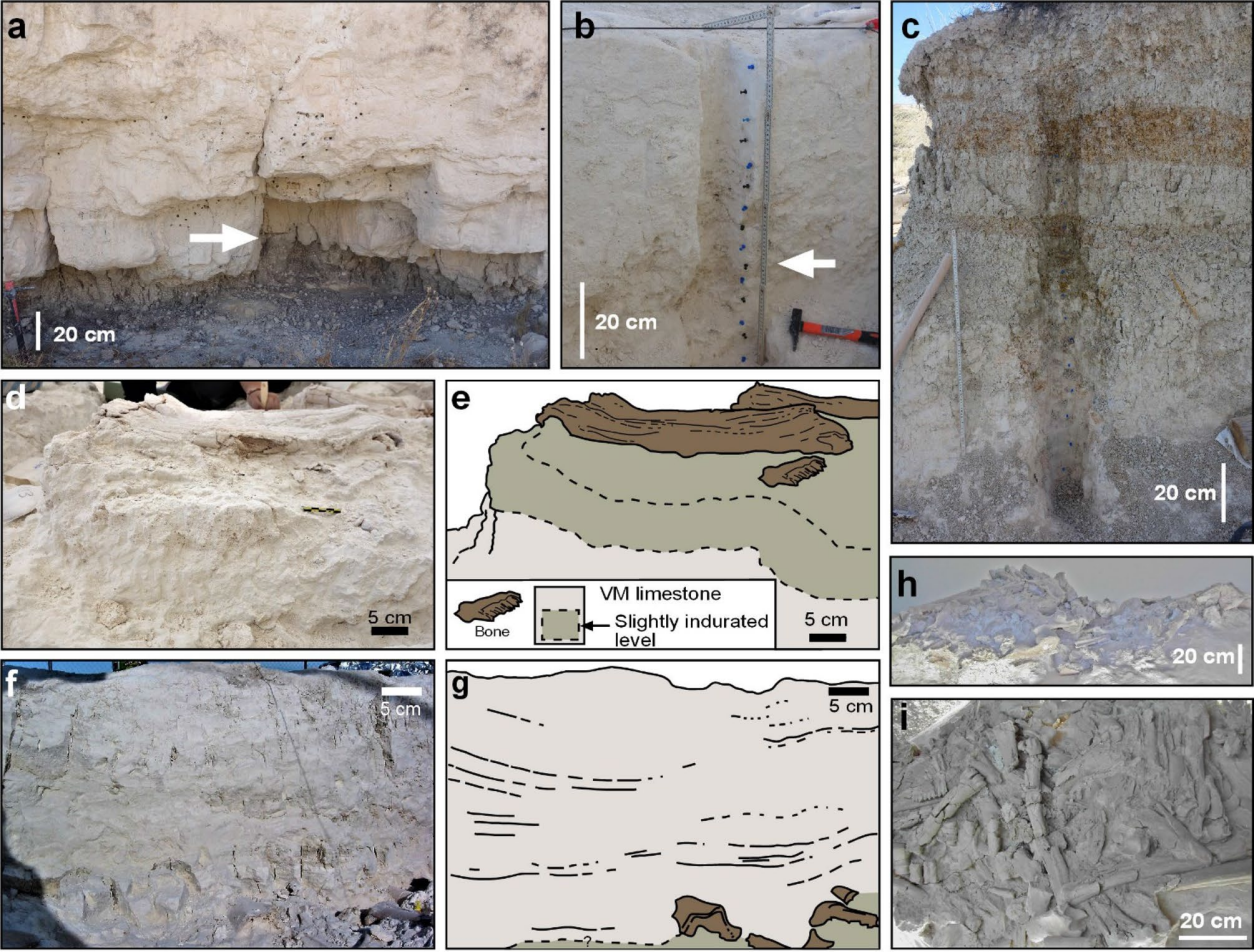
(**a**) Contact (arrow) between intervals C0 and C1 in section VM1. (**b**) Intervals C1 and C2 in section VM4 (arrow points to the limestone with subtle induration in Fig. 4b). (**c**) Unit D in section VM4. (**d**,**e**) Interval C1 in section VM4, showing large bones (a cervid horn, among others) on top of a level with subtle induration (also indicated in Fig. 1b). (**f**,**g**) Upper interval C1 and C2 in section VM3, displaying subtle stratification and lamination within C2. (**h**,**i**) Bone accumulation at site VM-3 in lateral and plane vertical view, respectively (as displayed at Orce Museum). Photographs (**a**–**d**) were taken by the authors in 2017, and (**f**–**i**) in 2005. The scale in Fig. a, h and i is approximate.

The VM site is an example of a bonebed^9^ where fossils appear minimally weathered or abraded, are commonly articulated (or associated) and display no evidence of tractive transport. On the basis of bone taphonomy and the overrepresentation of subadults, it has been suggested that such bone accumulation took place in a hyena den^10–12^. The mammalian fossil bones at the site represent a combination of species living in several environments, such as an open shrub environment (e.g., horses, the dominant group) and aquatic settings (e.g., hippopotamuses). The VM site is a cluster of several close-by localities (see Fig. 2), located 1 km to the west of Venta Micena village. Since its discovery in 1976, research at VM has yielded a wealth of mammalian taxonomic and taphonomic findings, mainly from site number 3 (VM3). Sites VM3 and VM4 (see Fig. 2) are the richest in large fossil bones and have been extensively excavated. Bones are found within a white micritic limestone (VM limestone) having a minimum lateral extent of 1 km and a thickness of around 1.5 m. The VM limestone is found between more detrital strata (Fig. 3a,c) and at first glance has a general massive aspect (Fig. 3b). In detail, it contains several diffusely stratified levels (Fig. 3f,g) that show very subtle indurated depressions (Fig. 3d,e) where bones should be accumulated (Fig. 3d,e,h,i)^13,14^.

The geological and paleolimnological context of the site was part of a larger study^15^ characterizing the lacustrine and palustrine environments of the VM limestone-bearing succession based on detailed data on lithostratigraphy, ostracods and other invertebrates^16^. Unfortunately, no further significant sedimentary research has been carried out at the VM sites, and only incompletely documented investigations^16,17^ have been executed. Regarding isotopic studies on invertebrates or rock samples from VM limestone, only three isolated measurements have been published^15^. Here, for the first time, we report an integrated geochemical and sedimentological study on VM limestone to characterize the paleoenvironmental conditions linked to the bone accumulation processes.

## Geological setting

The Guadix-Baza intramontane basin is located in the Betic Chain, at the contact between the internal and external units (Paleozoic to Triassic basement and external Mesozoic cover, respectively, see Fig. 1a). This basin was disconnected from the sea by 8 Ma^18^ and became an endorheic basin until it was captured by the Guadiana Menor river^19^ before 205 ka^20^. The two most important formations of the basin infill are the alluvial Guadix Formation^21^ and the Baza Formation (Baza Fm)^22^, characteristic of the Guadix and Baza sub-basins, respectively. The Baza Fm has a maximum thickness of over 100 m and is widely known for hosting Plio-Pleistocene mammalian sites^1,3^.

In the Galera-Orce-Fuente Nueva area (containing the VM site), up to three subunits (members) can be distinguished within the Baza Fm^23^. The younger Upper Member is mainly composed of lacustrine–palustrine carbonates with common paleosols. Salinity changes in these rocks have been investigated through analysis of the invertebrate faunas, mainly ostracods^24–26^. VM limestone^15,25^ has a carbonate content ranging between 80 and 100% for limestones and dolostones, between 60 and 79% for muddy limestones and muddy dolostones, and between 40 and 59% for calcareous mudstones and dolomitic mudstones. In the VM area, six units were described^25^, named as A to F. Note that Unit C is further subdivided into three intervals. Here, we review units A to E (see also Fig. 2):

- Unit A (4 m thick) contains dolomitic marls and micritic dolostones locally interbedded with gravel, sand, limestone and shale, the latter in finer strata. Root bioturbation is common and sandy levels display large-scale cross-stratification and ripple cross-lamination. This unit records a period of important subaerial exposure based on (a) a relatively poor content of invertebrates, since most of them have been dissolved by weathering (some moulds are visible), and (b) the presence of pedogenic features.
- Unit B (0.3 to 1.5 m) consists of bioclastic sands, marls and mudstones containing foraminifera, gastropods and ostracods. The unit is interpreted as representing a saline lake transgression episode on the basis of the presence of ostracods *Cyprydeis torosa* and the mollusc *Cerastoderma,* which live in waterbodies with salinities between 4‰ and 60‰^25^.
- Unit C (2.3 to 3.4 m) has a lower part (here named interval C0) with whitish sandy micritic limestone that is locally greenish and blackish (such as in section VM1, Fig. 2), and a micritic upper part, known as VM limestone. This upper limestone contains the paleontological sites and has a thickness of around 1.5 m. Generally, no invertebrate fauna is found in unit C, except in some sections such as section VM1, containing ostracods, gastropods (or their opercula) and charophyte gyrogonites. In VM limestone, two intervals (C1 and C2) can be differentiated on the basis of their bulk-rock isotopic values (see later). The lower half of the VM limestone (interval C1) contains fossil bones, with more than 50 elements per square metre, while the upper half (C2) is rather poor in fossil bones. It was stated^16^ that the bones rest on the relief of a paleosol, indicating that they accumulated during a subaerial exposure phase that was followed by micritic mud deposition.
- Unit D (7 m) contains muddy limestones interbedded with sandy marls, dolostones and marly dolostones (Fig. 3c). Some levels in this unit are reddish and brownish and show traces of root bioturbation.
- Unit E (7.5 m) is a succession of calcareous-dolomitic marls and marly-sandy dolostones that alternate with levels of sands and gravels with cross-stratification.

**Figure 4:**
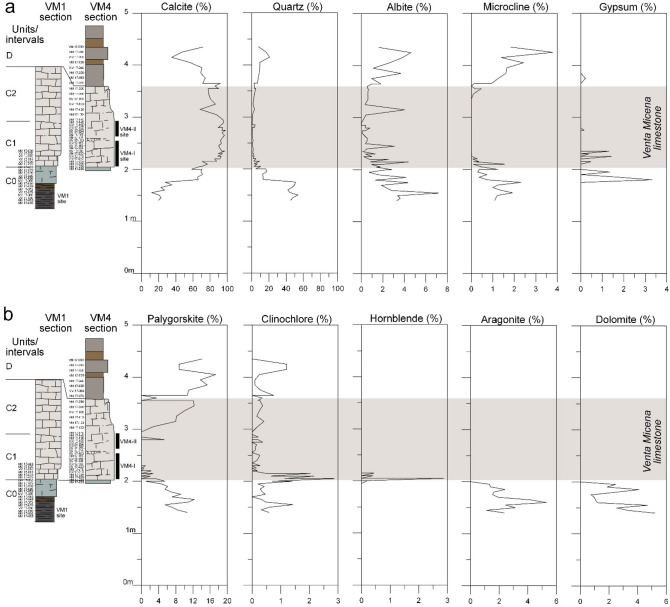
XRD results from composite VM4 and VM1 sections. (**a**) Percentages for calcite, quartz, albite, microcline and gypsum. (**b**) Percentages for palygorskite, clinochlore, hornblende, aragonite and dolomite. See values in STab. 1.

We review relevant sections containing units B to D (see Fig. 2) in order to avoid nomenclature confusion between sites and sections. First, section VM1 (from left to right) was designated as ‘ZM’ or ‘Barranco de los Zagales section’ in previous studies^15,25,26^, and the base of the section (interval C0) contains the site VM1^27^. This site essentially contains microvertebrates and is not considered part of the VM bonebed (mainly located in interval C1, but also in C2). Second, section VM4 in Fig. 2 contains the ‘Venta Micena 4’ site (here labelled as the VM4 site), which is probably referred to as ‘I’^12^. The latter was a natural outcrop, while section VM4 is an artificial trench. Third, section VM2 in Fig. 2 is referred to as ‘Sondeo 2′^12^, and is also the only locality where the Venta Micena limestone has yielded significant rodent faunas^3^. Fourth, section VMX in Fig. 2 was partially described as the ‘Cueva de los Champiñones section’ and ‘X’ profile^15,25,26^. Finally, section VM3 in Fig. 2 contains the ‘Venta Micena 3’ site and is part of the composite section ‘CT’^15,25,26^ (‘Cueva de Tomas’, 50 m to the north of site VM3; see Fig. 4a,c).

The investigation of ostracods^25^ determined salinity variations throughout the VM area, mainly in the CT profile. This work detected recurrent changes in water salinity, regime and solute composition, with an alternation between slightly saline, bicarbonate-rich waters and saline waters dominated by Na and Cl. A highly contrasted climate was detected, resembling that in the Mediterranean region today, but with a greater availability of water in the system if compared with the present-day situation in southern Spain. For unit B, data on ostracods indicated a non-marine high salinity phase (Na Cl), with abundant smooth-shelled *Cyprideis*. For unit C, no data were obtained in section CT, since no invertebrate fossils were found. Alternatively, unit C has been studied in section VM1, where coarsely reticulated and heavily noded *Cyprideis* indicate lower salinities (bicarbonate dominance, usually well below 10‰). After analysing the Sr/Ca and Mg/Ca ratios of ostracods, it was concluded^26^ that there was no marine influence, even for the high salinity intervals.

## Results

**Figure 5:**
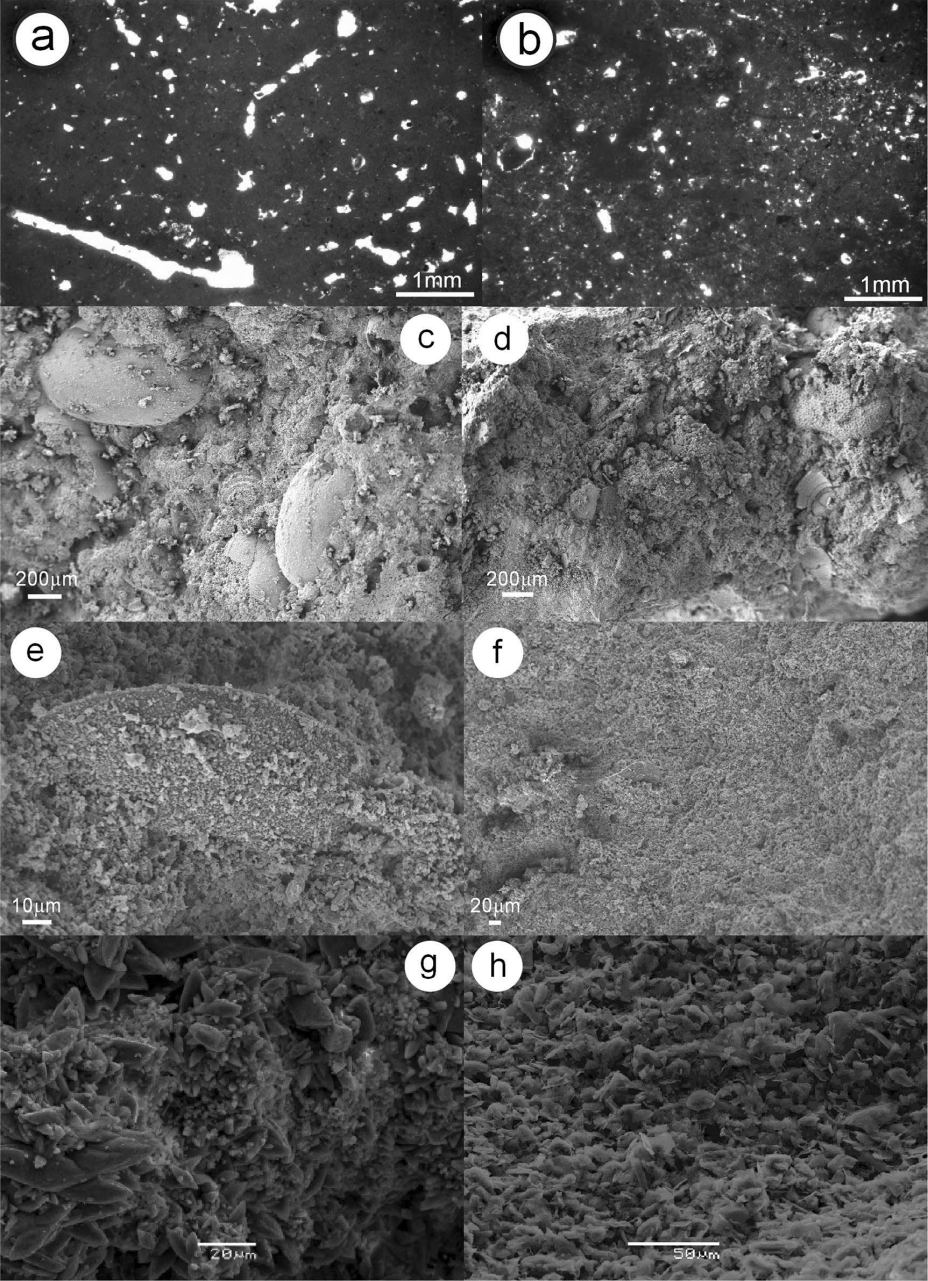
Petrography and SEM of the Venta Micena limestone. (**a**) Thin section of the lower subtly indurated level in the C1 interval in section VM4 (Fig. 3d,e). (**b**) Thin section of the subtly indurated level in Fig. 3b (C1 interval in section VM4). Both (**a**,**b**) show porosity likely to result from biogenic activity. (**c**,**d**) SEM images from section VM1, with abundant biogenic carbonates such as ostracods (intervals C1 and C2, respectively). (**e**,**f**) SEM images from section VM4 (interval C1), showing its general absence of invertebrates (**f**), except for rare microsparitized shells (an example of an ostracod test in **e**). (**g**,**h**) SEM images from the stratigraphic height 0.9 m in section VM3 (interval C1), with well-crystallized calcite scalenohedra (**g**), and the limestone interval between indurated levels 1 and 2 in section VM3 (**h**).

**Figure 6:**
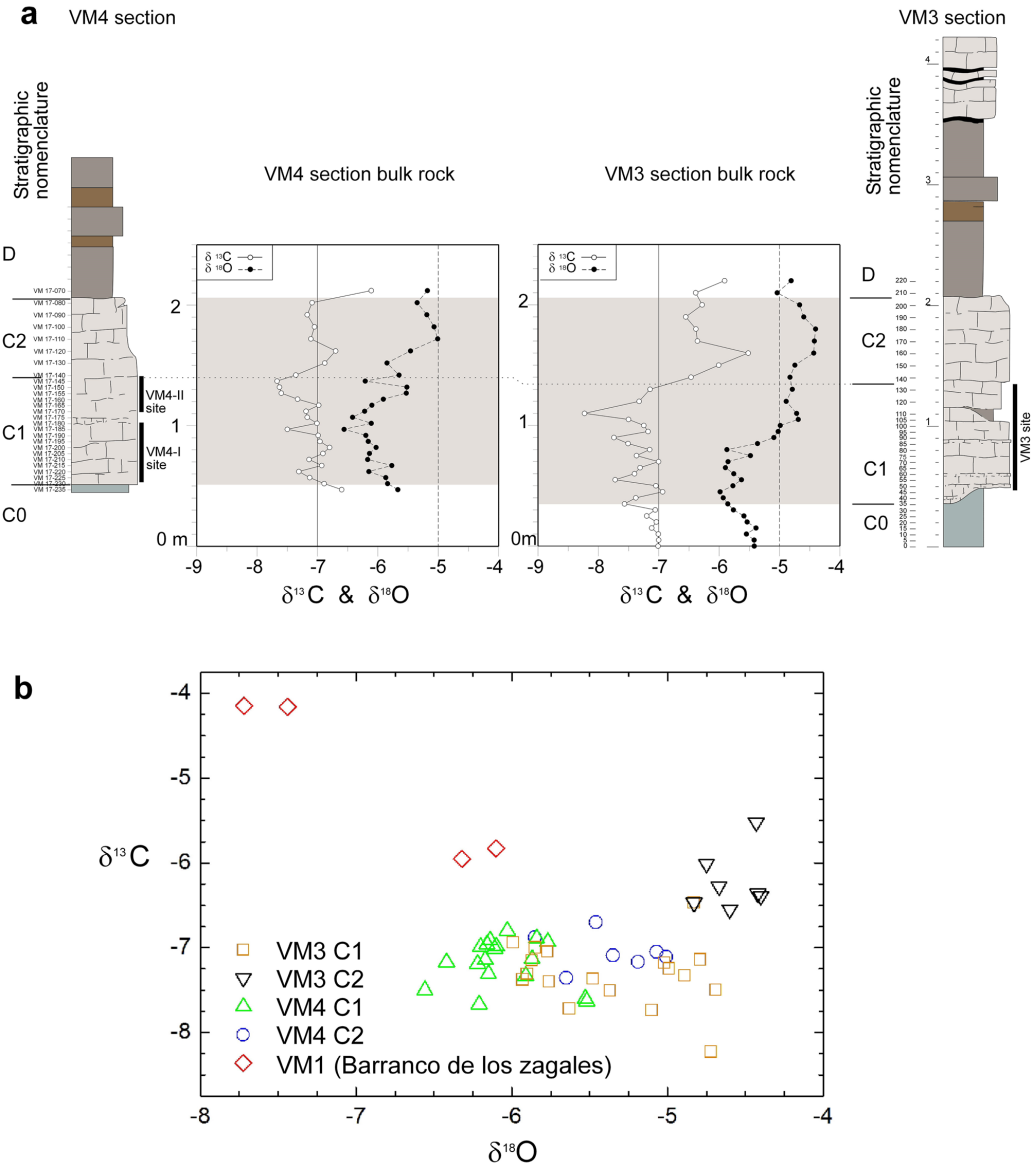
(**a**) Bulk-rock oxygen and carbon curves from sections VM3 and VM4. (**b**) Stable isotope cross-plots for sections VM3 and VM4. See values in STab. 3.

**Figure 7:**
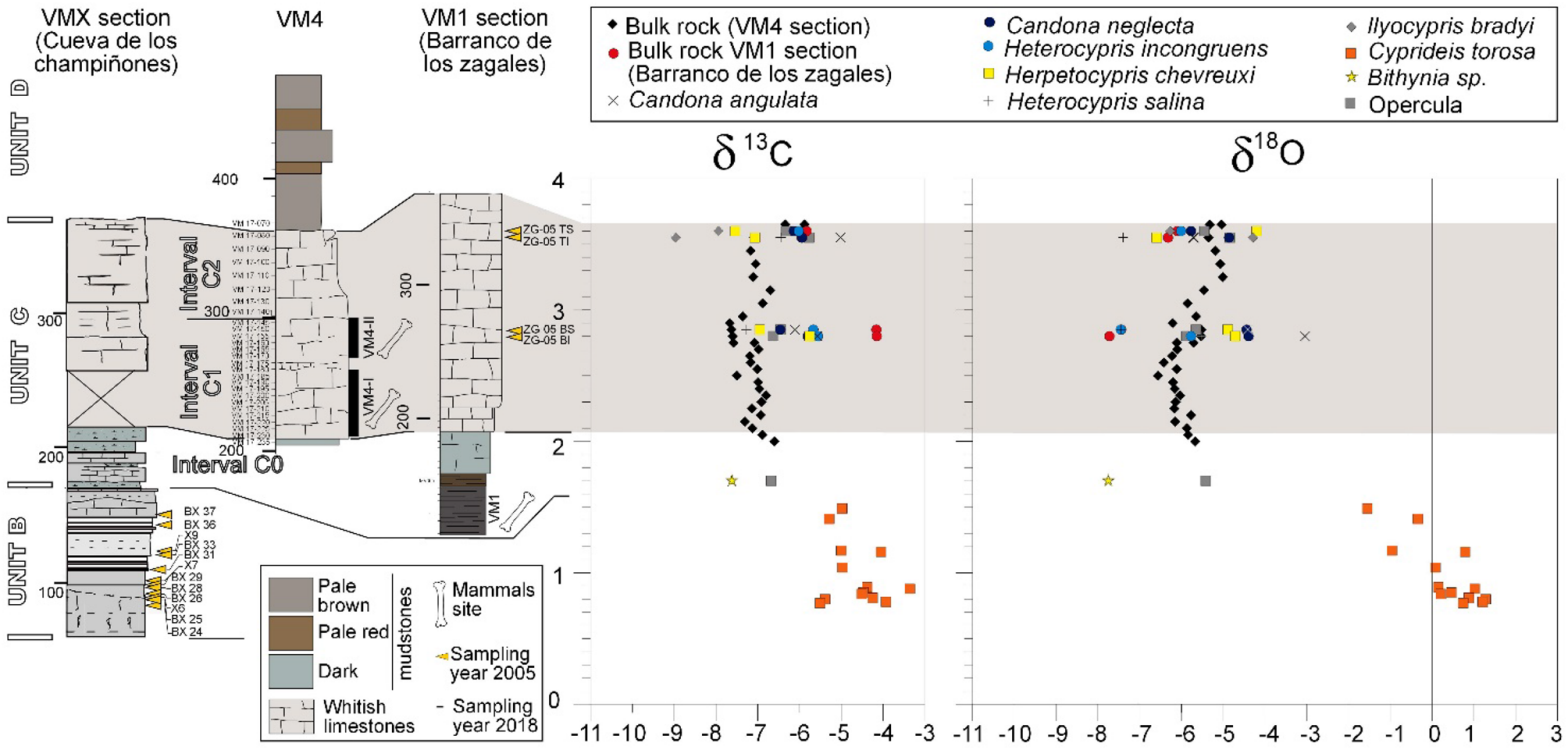
Isotopic stratigraphy of intervals B and C, containing bulk data from sections VM4 and VM1 and invertebrate data from VM1 and VMX. No invertebrate fauna was found from section VM4. See values in STab. 2 and STab. 3.

The analyses performed on the samples examined in this work (see applied methods in the Supplementary Information) yielded the following results (see Figs. 4 to 7):

### X-ray diffraction (XRD) mineralogy

XRD data were obtained from the lower section VM1 (interval C0) and from section VM4 (intervals C1 and C2, see later). The whole dataset is presented in Fig. 4 as a composite succession (see also Supplementary table STab. 1). This succession shows that carbonates such as calcite (Fig. 4a), aragonite and dolomite (Fig. 4b) and quartz (Fig. 4a) are the major components. VM limestone (C1 and C2) is very rich in low-Mg calcite, with values being between 70% and almost 100%. Interval C0 and unit D are richer in quartz and other clastics compared to C1 and C2. The mineralogical content also indicates that the contact between these clastic-rich spans (interval C0 and unit D) and VM limestone is transitional.

Minerals such as gypsum (Fig. 4a), aragonite and dolomite (Fig. 4b) are constantly below 6% and are only significant in C0 and lower C1. Along with major minerals, variable amounts of up to twenty minor constituents were detected, including the clay minerals palygorskite and kaolinite, between sample VM 17–000 and sample VM 17–150 (STab. 1). These represent products of mineral alteration or external contributions.

XRD performed on section VM1 also demonstrated an increase in the calcite content from the base to the top. The highest contents of calcite in the equivalent levels of the carbonate of section VM1 stand out (MV 17–010 = 90.7%) in contrast to section VM4 (VM 17–230 = 64.4%). Four samples from VM3 yielded similar results and demonstrated a mineral assemblage dominated by low-Mg calcite (between 92.5% and 97.9% for C1 samples; 77.6% for the C2 sample), along with quartz (2–17%) and minor amounts of dolomite (0.1–5%) and clay minerals (0.7–8.8%).

**Petrographic determinations.** Thin sections from section VM4 were extracted from two slightly indurated levels observed at the base and in the middle of interval C1 (Fig. 3b,d–e). Under the petrographic microscope (see Fig. 5a,b), a micropeloidal limestone was detected, with sparse small quartz grains and a fenestral porosity probably of organic origin (Fig. 5a). Detailed micromorphological study could provide further information to determine the origin of this porosity.

SEM imaging was very informative and provided straightforward determinations of two microfacies in VM limestone, one invertebrate rich and the other invertebrate poor, on the basis of aquatic skeletal remains of invertebrates and carbonate texture. Each microfacies is unrelated to C1 or C2 units, which are based on isotopic data. In addition to SEM imaging, the differentiation of these two microfacies was obvious during ostracod sieving and picking under a binocular microscope for isotope studies. The invertebrate-rich microfacies is dominant in section VM1 (Fig. 5c,d) and contains abundant bioclasts (tens per cm^3^), such as complete or fragmentary ostracod shells, as well as abundant charophyte gyrogonites. The matrix between these bioclasts is irregular and is built up from anhedral micritic-microsparite carbonate crystals of different sizes. Taxonomic analysis of the recorded species indicates sedimentation within a fresh or oligohaline water mass^15^.

Invertebrate-poor microfacies (Fig. 5e–h) were observed in samples from VM limestone in sections VM3 and VM4. After careful SEM observations, only very scarce ostracod moulds/replaced shells were detected (Fig. 5e). Essentially, the limestone is composed of micrite (less than 4 microns), microsparite (between 4 and 10 microns) and pseudosparite (between 10 and 50 microns). In general, subhedral or euhedral crystals can have variable sizes within a single sample (see Fig. 5g, belonging to a subtly indurated interval), but in other cases is rather homogeneous (Fig. 5h, corresponding to a barely lithified limestone). Well-developed crystals are far more abundant than in invertebrate-rich microfacies. Porosity is more evident in subtly indurated levels (as also seen in thin sections), which display initial crystallization of secondary calcite in the pore spaces as cement.

**Skeletal invertebrate isotopes.** Isotopic δ^18^O and δ^13^C analyses of the skeletal parts of invertebrates (see Methods S1, STab. 2 and Figs. 6 and 7) are used to infer paleohydrological conditions such as the isotopic concentration- dilution, temperature and dissolved carbon, although the latter is highly influenced by species metabolism and other ecological effects. The studied ostracods are extant species (see supplementary notes S2) with well-known ecological features and C and O isotopic vital offsets of the shells by rapport to the environment where they grow, so their isotopic signature is very useful for establishing environmental correlations from the fossil record. Such techniques have been applied to constrain the paleoenvironmental evolution recorded in the nearby Barranco León site^28^. Invertebrate remains for interval C turned out to be absent from sections VM3 and VM4, and they were consequently investigated in the nearby section VM1, where biogenic skeletal carbonates are abundant. Data from section VMX (Cueva de los Champiñones) are also included with the aim of obtaining a record from unit B.

The δ^18^O of the calcite from the ostracod valves is a function of the temperature of the water where ostracods moulted and the oxygen stable isotopic composition of the water. δ^18^O results from ostracod and gastropod opercula were normalized (corrected) by summing the vital fractionation and the values obtained in the analyses. According to the compilation in Ref.^29^, the correction factors are + 2.4‰ for *Candona neglecta,* + 2.2‰ for *Candona angulata,* + 0.8‰ for *Cyprideis torosa* and + 3.9‰ for *Ilyocypris bradyi.* For the opercula of *Bithynia* sp., the normalization factor would be 1.2‰, but for the δ^18^O data on gastropod aragonitic shells (*Bithynia*), no correction was applied, since it is usually observed^30–32^ that the shells reflect the oxygen conditions of the environment (i.e., are in isotopic equilibrium).

The δ^13^C value is an approximation to the isotopic composition of the dissolved inorganic carbon in the water. The δ^13^C signal in each individual is directly related to the fractionation of each species, even in different ontogenetic stages. In unit B (sampled in section VMX), *Cyprideis torosa* yielded values ranging between -5.51‰ and -3.93‰ for δ^13^C and between -1.55‰ and + 1.28‰ for δ^18^O.

The general negative trend, particularly for O values, indicates that the system evolved progressively to less concentrated waters. On the other hand, C and O values in section VM1 are rather different. See Fig. 7 and STab. 2 for details. In summary, unit C yielded more negative isotope values compared to those of unit B, especially for the O isotopes.

In section VM3, unit E levels located 7.5 to 10 m above the VM limestone contain *C. torosa* and *Ammonia beccarii*^28^ (P. Anadón and M. Gabàs, unpublished data, see also Supplementary notes S2), indicating that they formed in saline waters. Samples from three beds at the top of this interval display *C. torosa* shells with δ^13^C from − 6.41‰ to − 3.42‰ and δ^18^O from − 1.66 to 0.13‰ (P. Anadón and M. Gabàs, unpublished data). These features suggest that these levels formed in saline and isotopically more concentrated waters for δ^18^O.

At VMX (50 m to the S of VM3), the lowermost levels (interval A, see Supplementary Notes S2) contain freshwater ostracods and planorbid gastropods that exhibit depleted isotopic signatures (δ^13^C = − 9.64‰; δ^18^O = − 5.32‰). These beds are overlain by sandy carbonate mudstones (unit B) with a saline ostracod and mollusc fauna^15^. The isotopic signature of the *C. torosa* shells (BX samples in Figs. 2 and 7; n = 13) range from − 5.39‰ to − 3.93‰ in δ^13^C and from − 1.55‰ to 1.28‰ in δ^18^O (Anadón and Gabàs, unpublished).

Essentially, units A and B have not been recognized in other outcrops of the area. The paleoenvironmental features deduced from the invertebrate fossil content^15^ and the isotopic signatures of the biogenic skeletal carbonates indicate that interval A records an early freshwater wetland that was succeeded by a transgressive saline-lake phase recorded in unit B.

**Bulk-rock isotopes.** Bulk-rock samples from sections VM4 and VM3 (and also four samples from section VM1) were targeted for isotopic δ^13^C and δ^18^O analyses of the carbonate fraction (see S1 Methods and STab. 3). Clustering analysis of VM4 and VM3 data (see Fig. 6a and stratigraphically constrained similarity in Supplementary data S3) permitted the definition of intervals C1 and C2.

Section VM4 displayed δ^13^C bulk-rock isotope values (n = 27) ranging from − 7.67‰ to − 6.11‰, while δ^18^O values ranged from − 6.56‰ to − 5.01‰. Regression analysis of all the samples (Fig. 6b) did not reveal any significant correlation (r = 0.25). However, considering only the lower C1 interval of the VM limestone (samples VM17-235 to VM17-145 N, i.e., the lowermost 90 cm, n = 18), the values displayed no correlation (r = 0.14), although the variation in δ^13^C and δ^18^O is very low (around 1‰). The upper interval of the section (C2) (from samples VM17-140 N to VM17-80, n = 7) showed a correlation of r = 0.18. A general comparison between intervals C1 and C2 permitted the recognition of partial covariance in C1 (at least for some spans), a phenomenon not observed in interval C2.

Section VM3 samples (n = 34) (Fig. 6a) displayed bulk-rock δ^13^C values between a maximum of − 5.52‰ and a minimum of − 8.23‰ (VPDB), with a total range of 2.7‰. The majority of the δ^13^C data fell between − 6‰ and − 8‰; only two horizons (150 and 160 cm) showed slightly more enriched values (Fig. 6a,b), whilst only one level stood out with more depleted values (110 cm). The δ^18^O values of carbonates from section VM3 were relatively invariant, with values ranging from − 5.99‰ (40 cm) to − 4.40‰ (180 cm) and a total range of 1.6‰. The average section VM3 values for δ^13^C and δ^18^O were only weakly correlated (r = 0.48). However, when the δ^13^C and δ^18^O values of the carbonates were submitted to cluster analysis (Supplementary notes S-3), the two resulting zones defined between 0–130 cm and 140–220 cm displayed very low to low correlations (r = 0.25, n = 7 for C1 and r = 0.45, n = 17 for C2). The bulk-rock samples from section VM1 (n = 4) displayed δ^13^C values ranging from − 5.95‰ to − 4.15‰. δ^18^O values ranged from − 7.72‰ to − 6.10‰, displaying a correlation of r = − 0.977. Cluster analysis provides an independent criterion to split C1 from C2 (which is consistent with bone occurrence). A significant feature of the isotopic data is that considering the overall C1 samples from VM3 and VM4 (n = 35), a weak correlation between δ^13^C and δ^18^O is observed, and a significant correlation for C2 is attained (r = 0.73, n = 14).

## Discussion

Mineralogical XRD data from section VM4 (Fig. 4) indicate that interval C0 and unit D were sourced from the Internal Zones (Paleozoic basement, see Fig. 1)^19,21,22^. This means that during these intervals, the dimensions and connectivity of Guadix-Baza lake were large enough to permit siliciclastics from these distant zones to reach the VM area. On the other hand, the occurrence of in situ-formed carbonates in C1 and C2 indicates sedimentation in isolated and low-energy conditions. Notably, minerals such as aragonite, gypsum and dolomite are only found in interval C0, suggesting that waters were more concentrated than in unit D, in agreement with the salinity determinations^25^ pointing to less saline conditions during the sedimentation of VM limestone. This is supported by the paleoecology of ostracods and molluscs in section VM1, which indicates that these invertebrates thrived in freshwater or slightly saline waters, while saline species (*C. torosa*) are found in the underlying unit B^15,16^. Salinity variations based on the ostracod paleoecology of these sections^25^ are in agreement with the ostracod isotopic data presented here.

Petrological and SEM observations indicate that the invertebrate-rich microfacies (with well-preserved bioclasts in VM1) may have formed in an offshore position in a wetland/pond. This last setting would have no (or hardly any) aerial exposition, and persistent aquatic conditions would enhance the preservation of invertebrate shells. On the other hand, invertebrate-poor microfacies would result from episodes of emersion enhancing skeletal dissolution/recrystallization. These features indicate that they accumulated at the margin of a lake, where subaerial and subaquatic conditions coexisted. A contribution of travertine-like formation processes cannot be ruled out. In any case, the previously defined paleosols^17^ seem plausible in this scenario.

The general δ^13^C and δ^18^O values of bulk carbonate samples from sections VM3 and VM4 (Fig. 6) generally fall in the range of calcite formed from meteoric waters. In VM3 and VM4, an overall trend towards less negative values is observed. This feature, together with the weak and significant correlations between δ^13^C and δ^18^O (particularly in C2 over all samples from both sections), could reflect the evolution of a closed system, in which evaporation through time causes enrichment to more positive values. Although these trends are comparable to those from closed lakes that have been studied^33^, covariance is also a common feature of pedogenic calcites formed in arid environments. In this case, the enrichment trend of δ^18^O is due to kinematic effects related to evaporation^34^. The correlated δ^13^C enrichment is attained through decreasing respiration rates and CO_2_ degassing. The low variance in δ^18^O, however, could indicate that the time span of these hydrological conditions was relatively short. A more detailed study could shed light on this point. Interestingly, fossil accumulations at sites VM4 and VM3 were mainly found within this C1 interval. The interval C2 showed no correlation between δ^13^C and δ^18^O, indicating a hydrologically open system^33,34^. The general variability and trend of our dataset (Fig. 6b) is very similar to data obtained from palustrine limestones of a similar age in the central Guadix sub-basin^35^.

The generally more negative δ^13^C and δ^18^O values of C1 in sections VM3 and VM4 coincide with the layers of subtly indurated levels and bones. This, together with the SEM images illustrating a strong component of secondary calcite, suggests that sediments emerged and underwent attendant dissolution and reprecipitation of calcite, and consequently, carbonates partly reflect the isotopic compositions of the secondary, sparitic calcite, noticed in their more depleted values, i.e., greater influence of meteoric waters. In C2, in turn, such features are less developed (fewer bones and less subtly indurated levels). Therefore, C2 sediments have inherited more of their isotopic composition from the parental lacustrine carbonates. In summary, we correlate subaerial exposition, geochemistry and bone occurrence.

The paleoenvironmental features deduced from the invertebrate fossil content^15^ and the isotopic signatures of the biogenic skeletal carbonates presented here indicate that unit A records an early freshwater wetland that was succeeded by a transgressive saline-lake phase recorded in unit B (in VMX). Unit C, in VM1, represents a freshwater wetland/pond where carbonate deposition took place. The carbonate deposits (VM limestone) in the other sections of VM area represent deposits that experienced successive subaerial exposures and pedogenesis. The overlying sediments of units D and E record a new saline lake phase and isotopically concentrated waters, as recorded by fossils and the isotopic features.

**Figure 8:**
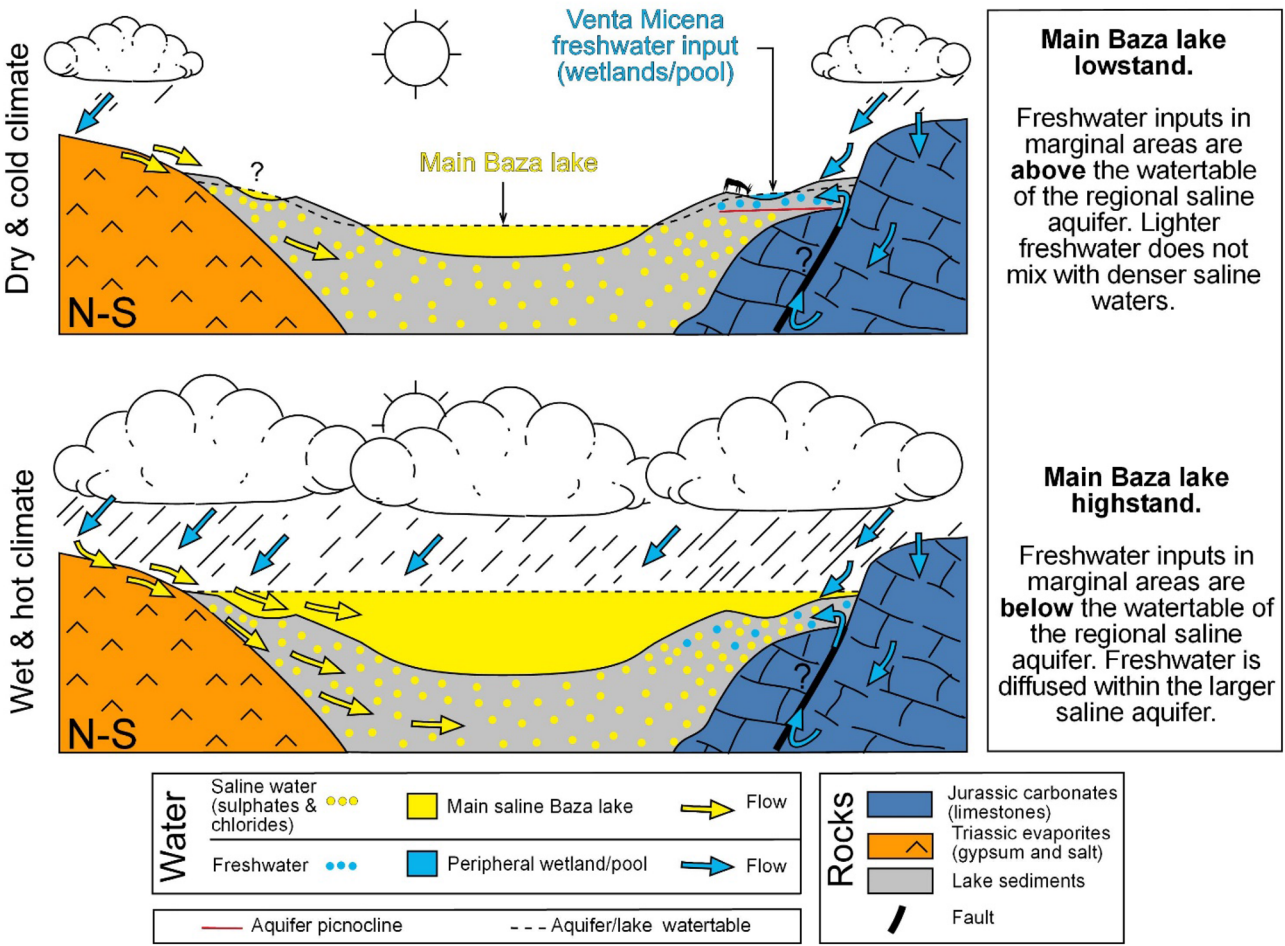
Evolutive hydrochemical model for the succession of the Venta Micena section. The lake highstand belongs to stratigraphic unit B (and also D), while the lake lowstand belongs to unit C (which contains the Venta Micena limestone paleontological sites). The likely input of thermal waters cannot be demonstrated in this study (see text). The vertical scale is exaggerated. The figure was created with Inkscape 1.0.1 (3bc2e813f5, 2020-09-07) software, https://inkscape.org/es/.

In a comparable isotope study^28^ in the nearby locality of Barranco León (BL)^36^, a similar sequence was found. Tentatively, the transgression unit at BL (levels A and B) could be correlated with interval B in the VM area (Fig. 7). This idea is supported by the fact that the VM sites are biostratigraphically older than the BL site^1^. Interestingly, the more detailed isotope data from BL (which documented the role of hydrothermal freshwater) support the model for the formation of VM sites suggested here. Although BL is a paleontological site that underwent reworking, a long-term model for bone concentration (or preconcentration) during lowstands is envisaged. The widespread occurrence of present-day hydrothermalism at the margins of the Guadix-Baza basin was used to explain the occurrence of vertebrate fossil sites^37^. This last model was debated, since freshwater environments were considered to be peripherical ones in the context of interconnected subenvironments^38^. Our data and model (in Fig. 8) are not only consistent in themselves, but also make these two last works compatible. That is to say, freshwater inputs can persist at relatively high margins of the basin and can evolve laterally (in terms of hydrochemistry and sedimentary facies) to the saline environments of the main lake.

## Conclusions

In summary, during the sedimentation of VM limestone: (1) there was hardly any clastic input, (2) sedimentation occurred during a freshwater interval separated by two more saline ones, as evidenced by ostracod paleoecology and the isotope signal, (3) this freshwater period had to take place during a lowstand, when the peripheric VM source was disconnected from the main saline Baza lake. Paradoxically, high rainfall would correspond to higher salinities in the VM area (a saline lake would concentrate freshwater inputs due to the lake highstand), while in drier periods (lake lowstand), the saline lake would not interfere with freshwater inputs. Regarding the conditions and isotope data of C1 (the interval concentrating bones), it is observed that (1) the central microfacies of VM1 contain no bones, while marginal (dominant) microfacies are isotopically more diluted, and (2) the shifts and partial oxygen covariance in C1 indicate that lake bones accumulated in short-lived intervals with closed hydrological conditions.

In terms of taphonomy, it is concluded that VM bones accumulated at the margin of a freshwater area that attracted mammals and their predators. Bones accumulated in situ in depressions that were flooded and buried by pond sediments due to water table pulsations. This paleoenvironmental setting also makes this scenario plausible for the activity of hyenas as a complementary factor for bone accumulation^10–13^.

## Supplementary Information

Supplementary Information.
