# Supplementary material for: Geochemical and sedimentary constraints on the formation of the Venta Micena early Pleistocene site (Guadix-Baza Basin, Spain)
Source: Sci Rep. 2021 Nov 17;11:22437. doi: 10.1038/s41598-021-01711-7 (PMC8599509; doi:10.1038/s41598-021-01711-7)
Supplement: Supplementary file 1 — Supplementary Information. [file 41598_2021_1711_MOESM1_ESM.docx]

**SUPPLEMENTARY INFORMATION**

**GEOCHEMICAL AND SEDIMENTARY CONSTRAINTS ON THE FORMATION OF THE VENTA MICENA EARLY PLEISTOCENE SITE (GUADIX-BAZA BASIN, SPAIN)**

Alejandro Granados et al.

Correspondence to: [joseporiol.oms@uab.cat](mailto:joseporiol.oms@uab.cat)

**This PDF file includes:**

**Supplementary Notes S1 – Page 1**

**Supplementary Notes S2 – Page 8**

**Supplementary Notes S3- Page 8**

**S1-GEOLOGICAL/GEOCHEMICAL METHODS**

**S1.1-Field sampling**

Sampling of sections VM-4 and VM-1 took place in July 2017 (A.G., O.O. and Víctor Fondevilla), while VM3 was sampled in 2018 (A.G.). Samples from VMX, VM1 and VM2 were collected in 2005 (P.A.). Sampling was performed together with sedimentary logging, and was generally conducted at intervals of 5 to 10 centimetres in fresh outcrops to avoid weathered materials. Samples from 70 levels were considered. Some of these samples were not analysed but may also appear labelled in the figures of the main text. The sample remains are stored at Universitat Autònoma de Barcelona (O.O.). Twenty samples provided biogenic carbonates that were isolated and analysed apart from the bulk rock. In section VM4, two paleosols were sampled to obtain thin sections. Five samples for micromorphology were also obtained, but are not part of this study. A total of 68 samples underwent XRD.

**S1.2 Sample preparation. Laboratory methodology**

Bulk rock

The laboratory work began by grinding the samples with the help of a hammer for the coarsest reduction, with the subsequent use of an agate mortar for homogenization to sixty microns. To avoid sample contamination, grinding elements were cleaned with distilled water at the end of the treatment of each sample. When using the hammer, samples were crushed within folded paper to avoid direct contact with the metal. Once samples had been homogenized, they were transferred to glass vials labelled with the same acronyms that were used in the field.

Milled subsamples underwent XRD and stable isotope studies. For the analysis of isotopes, we proceeded in advance to perform an analytical analysis of their composition by X-ray diffraction.

Skeletal biogenic carbonates

For stable isotope analyses on biogenic carbonates, the methodology followed that described in Anadón et al. (2015). The samples were soaked in a 6 vol% solution of H_2_O_2_ for 24 h, sieved with a 0.125-mm to 2-mm-mesh sieve and air-dried. Manual picking was carried out on the dried sieved samples for small fossil remains. For geochemical analyses, diverse biogenic skeletal carbonates were selected, including ostracod valves (calcite), aragonite mollusc shells and calcite opercula from *Bithynia*. Before the analysis, each biogenic piece was cleaned with a fine brush and a 96% ethanol solution. Mollusc shells were ground to a powder in an agate mortar, while the skeletal elements were directly analysed.

Thin sections were prepared in the laboratory of thin sections of the Geology Department at Universitat Autònoma de Barcelona.

**S1.3 SEM and microscope determinations**

Prior to SEM analyses, blocks of sediment were fractured to reveal a freshly exposed surface and mounted on SEM stubs using carbon conductive glue before vacuum sputter coating with gold.

SEM images from VM-4 and Barranco de los Conejos sections were acquired using a Zeiss EVO MA10 scanning electron microscope at Universitat Autònoma de Barcelona (Scientific-Technical Services), equipped with a W filament source and an Oxford Instruments x-act X-ray Silicon Drift Detector, which enables chemical microanalysis. Optical microscopes at the Geology Department of UAB were also used.

Eight samples from section VM-3 were selected for SEM examination. The secondary and backscattered electron images were taken using a Jeol JSM-5900LV instrument at the Geological Survey of Finland. The analytical conditions were as follows: low vacuum mode (22 Pa), a COMPO back-scattered signal, 20 kV accelerating voltage and 1 nA probe current.

**S1.4 Laboratory analysis**

The XRD analyses of VM-1 and VM3 were carried out at Geosciences Barcelona (GEO3BCN-CSIC). The equipment used was a Bruker D8-A25 diffractometer equipped with a Cu X-ray source (CuKα radiation) and a LynxEye position sensitive detector. Phase identification was carried out by using the software DIFFRAC.EVA in combination with the Powder Diffraction File (PDF-2) and the Crystallography Open Database (COD). Quantitative phase analyses were performed with the Rietveld method, using TOPAS 4.2 software from Bruker. These analyses allowed us to evaluate the crystallinity of calcite in Unit C of VM4 (see below).

Further determinations from VM3 were obtained with a PANalytical X’Pert3 Powder instrument with CuKα1 radiation in a focused beam geometry at the Department of Geosciences and Geography, University of Helsinki. Mineral identification was performed in Highscore Plus software utilizing the ICDD PDF-4 Minerals database. Estimation of phase abundances was carried out with Rietveld refinement using Highscore Plus software.

For the isotope analysis, the facilities of the University of Salamanca (Spain) were used in the Static Isotope Analysis Service. The procedure consisted of obtaining CO_2_ for the determination of isotopic ratios of ^13^C/^12^C and ^18^O/^16^O by reaction with 103% H_3_PO_4_ at 25 °C in the carbonate line. In samples of biogenic origin that contained very little material, sufficient gas was obtained for all determinations. The determination of isotopic ratios by gas source mass spectrometry was carried out in "Dual Inlet" mode using a SIRA-II spectrometer.

Carbon and oxygen isotopic ratios from section VM3 were measured with a Finnigan MAT253 mass spectrometer coupled with an online Gasbench II (reaction with water-free phosphoric acid at 45 °C) at the Vrije University, Amsterdam. The mass spectrometer was calibrated using the international standard IAEA-603 (δ^13^C +2.46‰ and δ^18^O -2.37‰). The standard deviation of the measurements for δ^13^C and δ^18^O was <0.1‰ and <0.2‰, respectively. Ratios of carbon and oxygen isotopes are given as parts per million (ppm) relative to the VPDB standard and expressed as δ values.

STab. 1 XRD quantification from sections VM 4, VM1 and VM 3 in order of descending height

| Height (m) | Sample | **Section** | **Calcite** | **Quartz** | **Albite** | **Microcline** | **Gypsum** | **Palygorskite** | **Illite** | **Kaolinite** | **Paragonite** | **Clinochlore** | **Hornblende** | **Aragonite** | **Dolomite** |
| --- | --- | --- | --- | --- | --- | --- | --- | --- | --- | --- | --- | --- | --- | --- | --- |
| 4.35 | VM17-000 | VM4 | 71.695 | 7.853 | 1.609 | 1.83 | 0 | 14.103 | 2.118 | 0.66 | 0.134 | 0 | 0 | 0 | 0 |
| 4.25 | VM17-010 | VM4 | 47.127 | 16.508 | 4.625 | 3.797 | 0 | 8.77 | 10.455 | 0.277 | 7.231 | 1.209 | 0 | 0 | 0 |
| 4.15 | VM17-020 | VM4 | 35.203 | 20.718 | 3.797 | 1.389 | 0 | 8.805 | 13.282 | 0.279 | 15.32 | 1.206 | 0 | 0 | 0 |
| 4.05 | VM17-030 | VM4 | 56.804 | 9.494 | 2.577 | 2.414 | 0 | 17.273 | 3.683 | 0.868 | 6.638 | 0.249 | 0 | 0 | 0 |
| 3.95 | VM17-040 | VM4 | 70.382 | 9.349 | 1.085 | 1.645 | 0 | 13.717 | 3.425 | 0.358 | 0.002 | 0.096 | 0 | 0 | 0 |
| 3.85 | VM17-050 | VM4 | 69.004 | 8.759 | 3.668 | 1.618 | 0 | 15.338 | 1.104 | 0.38 | 0 | 0.092 | 0 | 0 | 0 |
| 3.75 | VM17-060 | VM4 | 74.347 | 7.506 | 0.968 | 1.258 | 0.212 | 10.956 | 3.889 | 0.31 | 0.266 | 0.288 | 0 | 0 | 0 |
| 3.65 | VM17-070 | VM4 | 71.399 | 6.706 | 1.818 | 0.93 | 0 | 10.708 | 4.074 | 0 | 3.612 | 0.757 | 0 | 0 | 0 |
| 3.65 | VM17-070N | VM4 | 91.921 | 2.595 | 0.985 | 0.126 | 0 | 0 | 3.46 | 0 | 0.913 | 0 | 0 | 0 | 0 |
| 3.60 | ZG-05 TS | VM1 | 85.572 | 3.243 | 0.558 | 0 | 0 | 3.529 | 3.805 | 0.141 | 2.807 | 0.344 | 0 | 0 | 0 |
| 3.55 | ZG-05 TI | VM1 | 89.736 | 3.052 | 0.602 | 0.174 | 0 | 0 | 4.58 | 0 | 1.594 | 0.2 | 0 | 0 | 0 |
| 3.55 | VM17-080 | VM4 | 77.829 | 4.633 | 0.655 | 0.456 | 0 | 12.086 | 2.922 | 0.302 | 0.846 | 0.27 | 0 | 0 | 0 |
| 3.45 | VM17-090 | VM4 | 78.24 | 3.28 | 0.595 | 0.092 | 0 | 12.275 | 3.744 | 0 | 1.404 | 0.364 | 0 | 0 | 0 |
| 3.35 | VM17-100 | VM4 | 80.303 | 2.286 | 0.62 | 0 | 0 | 9.933 | 4.696 | 0.318 | 1.59 | 0.245 | 0 | 0 | 0 |
| 3.25 | VM17-110 | VM4 | 86.206 | 1.408 | 0.334 | 0 | 0 | 8.154 | 3.061 | 0.178 | 0.464 | 0.16 | 0 | 0 | 0 |
| 3.15 | VM17-120 | VM4 | 68.431 | 3.235 | 4.047 | 0 | 0 | 7.996 | 10.221 | 0.252 | 5.407 | 0.41 | 0 | 0 | 0 |
| 3.05 | VM17-130 | VM4 | 87.215 | 0.909 | 0.423 | 0 | 0 | 2.816 | 5.463 | 0 | 2.982 | 0.189 | 0 | 0 | 0 |
| 2.95 | VM17-140 | VM4 | 95.006 | 0.997 | 0.151 | 0 | 0 | 0 | 2.339 | 0 | 1.507 | 0 | 0 | 0 | 0 |
| 2.90 | VM17-145 | VM4 | 95.957 | 0.613 | 0 | 0 | 0 | 0 | 2.653 | 0 | 0.776 | 0 | 0 | 0 | 0 |
| 2.85 | VM17-150 | VM4 | 93.053 | 0.707 | 0 | 0 | 0 | 0 | 3.714 | 0 | 2.317 | 0.208 | 0 | 0 | 0 |
| 2.85 | ZG-05 BS | VM1 | 92.893 | 4.01 | 0.401 | 0 | 0 | 0 | 2.251 | 0 | 0.308 | 0 | 0 | 0 | 0 |
| 2.80 | ZG-05 BI | VM1 | 89.288 | 3.165 | 0.408 | 0 | 0 | 5.315 | 0.892 | 0.11 | 0.718 | 0 | 0 | 0 | 0 |
| 2.80 | VM17-155 | VM4 | 94.61 | 0.436 | 0.782 | 0 | 0 | 0 | 2.44 | 0 | 1.534 | 0.198 | 0 | 0 | 0 |
| 2.75 | VM17-160 | VM4 | 93.692 | 1.106 | 0.399 | 0 | 0.153 | 0 | 3.275 | 0.052 | 0.935 | 0.372 | 0 | 0 | 0 |
| 2.75 | VM17-160N | VM4 | 97.87 | 0.64 | 0.124 | 0 | 0 | 0 | 1.05 | 0 | 0 | 0 | 0 | 0 | 0 |
| 2.70 | VM17-165N | VM4 | 93.75 | 0.561 | 0.079 | 0 | 0 | 0 | 4.809 | 0 | 0.592 | 0.154 | 0 | 0 | 0 |
| 2.65 | VM17-170 | VM4 | 96.662 | 0.701 | 0.306 | 0 | 0 | 0 | 2.224 | 0 | 0 | 0.102 | 0 | 0 | 0 |
| 2.60 | VM17-175 | VM4 | 94.123 | 0.988 | 0.558 | 0 | 0 | 0 | 2.735 | 0 | 1.487 | 0.109 | 0 | 0 | 0 |
| 2.55 | VM17-180 | VM4 | 91.95 | 1.516 | 0.538 | 0 | 0 | 0 | 4.057 | 0 | 1.859 | 0 | 0 | 0 | 0 |
| 2.50 | VM17-185 | VM4 | 93.187 | 1.049 | 0.376 | 0 | 0 | 0 | 3.003 | 0 | 2.011 | 0.263 | 0 | 0 | 0 |
| 2.45 | VM17-190 | VM4 | 89.959 | 0.853 | 3.111 | 0 | 0 | 0 | 3.732 | 0 | 2.05 | 0.224 | 0 | 0 | 0 |
| 2.40 | VM17-195 | VM4 | 91.371 | 1.532 | 0.506 | 0 | 0 | 0 | 4.368 | 0 | 1.993 | 0.163 | 0 | 0 | 0 |
| 2.35 | VM17-200 | VM4 | 93.419 | 1.577 | 0.239 | 0 | 0 | 0 | 4.039 | 0 | 0.604 | 0 | 0 | 0 | 0 |
| 2.35 | MV17-000 | VM1 | 97.182 | 1.338 | 0.073 | 0.005 | 1.313 | 0 | 0 | 0 | 0 | 0 | 0 | 0 | 0 |
| 2.30 | VM17-205 | VM4 | 89.239 | 1.295 | 1.315 | 0 | 0 | 0 | 5.771 | 0 | 2.086 | 0.272 | 0 | 0 | 0 |
| 2.30 | MV17-005 | VM1 | 94.323 | 2.258 | 0.391 | 0.017 | 0.379 | 0.944 | 1.687 | 0 | 0 | 0 | 0 | 0 | 0 |
| 2.25 | VM17-210 | VM4 | 88.827 | 1.013 | 0.993 | 0 | 0 | 0 | 5.605 | 0 | 3.281 | 0.147 | 0 | 0 | 0 |
| 2.25 | MV17-010 | VM1 | 92.826 | 2.814 | 0.208 | 0.025 | 1.439 | 0.592 | 2.052 | 0 | 0 | 0 | 0 | 0 | 0 |
| 2.20 | VM17-215 | VM4 | 91.574 | 1.379 | 0.747 | 0.284 | 0 | 0 | 3.606 | 0 | 2.186 | 0.185 | 0 | 0 | 0 |
| 2.20 | MV17-015 | VM1 | 85.111 | 4.602 | 2.558 | 0.058 | 0 | 2.279 | 3.047 | 2.189 | 0 | 0 | 0 | 0 | 0 |
| 2.15 | VM17-220 | VM4 | 87.543 | 2.489 | 0.851 | 0.345 | 0 | 0 | 6.119 | 0 | 2.093 | 0.483 | 0 | 0 | 0 |
| 2.15 | MV17-020 | VM1 | 70.778 | 9.152 | 4.388 | 0.091 | 0.479 | 2.737 | 2.388 | 2.312 | 5.198 | 2.033 | 0.443 | 0 | 0 |
| 2.10 | VM17-225 | VM4 | 76.803 | 2.792 | 1.848 | 1.556 | 0 | 0 | 11.226 | 0.179 | 4.937 | 0.657 | 0 | 0 | 0 |
| 2.10 | MV17-025 | VM1 | 77 | 6.225 | 0.954 | 0.09 | 0 | 2.132 | 3.489 | 2.633 | 4.917 | 2.16 | 0.4 | 0 | 0 |
| 2.05 | VM17-230 | VM4 | 69.858 | 3.024 | 1.788 | 0.458 | 0 | 0 | 14.77 | 0.234 | 8.737 | 1.131 | 0 | 0 | 0 |
| 2.05 | MV17-030 | VM1 | 67.817 | 10.475 | 1.361 | 0.429 | 0 | 3.318 | 4.119 | 2.516 | 6.524 | 2.882 | 2.882 | 0 | 0 |
| 2.00 | VM17-235 | VM4 | 58.313 | 4.63 | 2.85 | 1.24 | 0 | 5.519 | 14.931 | 0.567 | 11.253 | 0.696 | 0 | 0 | 0 |
| 2.00 | MV17-035 | VM1 | 70.528 | 16.479 | 2.415 | 0.373 | 0 | 1.027 | 0.922 | 1.656 | 5.007 | 1.405 | 0.188 | 0 | 0 |
| 1.95 | MV17-040 | VM1 | 69.272 | 17.532 | 0.71 | 0.296 | 1.354 | 4.689 | 0.569 | 0 | 1.601 | 0.191 | 0 | 1.33 | 2.457 |
| 1.90 | MV17-045 | VM1 | 71.283 | 12.48 | 1.628 | 0.724 | 0 | 6.101 | 0.971 | 0 | 3.138 | 0.422 | 0 | 1.392 | 1.86 |
| 1.85 | MV17-050 | VM1 | 65.485 | 13.34 | 4.195 | 0.364 | 1.767 | 5.445 | 1.034 | 0 | 1.557 | 0.283 | 0 | 2.409 | 4.123 |
| 1.80 | MV17-055 | VM1 | 65.988 | 12.99 | 1.29 | 0.73 | 3.327 | 6.732 | 1.864 | 0 | 2.689 | 0.344 | 0 | 1.62 | 2.426 |
| 1.75 | MV17-060 | VM1 | 27.808 | 50.869 | 4.363 | 2.311 | 0 | 9.117 | 0.817 | 0.639 | 1.305 | 0.452 | 0 | 1.554 | 0.766 |
| 1.70 | MV17-065 | VM1 | 35.525 | 50.112 | 1.886 | 1.243 | 0 | 7.16 | 0.165 | 0.928 | 0 | 0 | 0 | 1.94 | 0.983 |
| 1.65 | MV17-070 | VM1 | 22.682 | 47.187 | 2.321 | 1.002 | 0 | 12.25 | 4.133 | 0.839 | 4.005 | 0.322 | 0 | 4.184 | 1.075 |
| 1.60 | MV17-075 | VM1 | 26.875 | 42.054 | 2.824 | 1.365 | 0 | 10.964 | 4.169 | 0.861 | 3.909 | 0.477 | 0 | 5.31 | 1.193 |
| 1.55 | MV17-080 | VM1 | 12.076 | 44.137 | 7.201 | 1.922 | 0 | 5.438 | 10.476 | 0.59 | 9.593 | 1.426 | 0 | 2.437 | 4.703 |
| 1.50 | MV17-085 | VM1 | 21.121 | 53.778 | 3.391 | 1.214 | 0 | 7.15 | 3.777 | 0.694 | 2.618 | 0.589 | 0 | 3.136 | 2.534 |
| 1.45 | MV17-090 | VM1 | 22.619 | 47.891 | 3.62 | 1.137 | 0 | 8.328 | 5.186 | 0.896 | 5.534 | 0.319 | 0 | 1.095 | 3.376 |
| 1.40 | MV17-095 | VM1 | 19.74 | 45.315 | 3.283 | 1.081 | 0 | 10.72 | 6.158 | 0.751 | 4.776 | 0.593 | 0 | 2.363 | 5.219 |

STab. 2. Isotopic values for biogenic carbonates (sections VM1 and VMX). Samples ‘ZG’ and ‘MV’ belong to section VM1, while samples ‘BX’ and ‘X’ belong to section VMX.

| Height (m) | Sample | gastropod opercula | | *C. angulata* | | *C. neglecta* | | *H. salina* | | *H. incongruens* | | *H. chevreuxi* | | *C. torosa* | | *I. bradyii* | | *Bythinia* shell | |
| --- | --- | --- | --- | --- | --- | --- | --- | --- | --- | --- | --- | --- | --- | --- | --- | --- | --- | --- | --- |
|  |  | δ 13C | δ 18O | δ 13C | δ 18O | δ 13C | δ 18O | δ 13C | δ 18O | δ 13C | δ 18O | δ 13C | δ 18O | δ 13C | δ 18O | δ 13C | δ 18 | δ 13C | δ18O |
| 3.60 | ZG-05 TS | -6.33 | -5.45 |  |  | -6.14 | -5.77 |  |  | -6.02 | -6.01 | -7.54 | -4.20 |  |  | -7.94 | -6.27 |  |  |
| 3.55 | ZG-05 TI | -5.77 | -4.84 | -5.02 | -5.71 | -5.94 | -4.86 | -6.45 | -7.40 |  |  | -7.07 | -6.59 |  |  | -8.96 | -4.29 |  |  |
| 2.85 | ZG-05BS | -6.45 | -5.65 | -6.11 | -4.41 | -6.46 | -4.44 | -7.27 | -7.45 | -5.67 | -7.45 | -6.95 | -4.89 | -9.75 | -10.2 |  |  |  |  |
| 2.80 | ZG-05 BI | -6.64 | -5.88 | -5.57 | -3.04 | -5.8 | -4.39 | -5.79 | -5.51 | -5.56 | -5.77 | -5.76 | -4.71 |  |  |  |  |  |  |
| 1.70 | MV17-065 | -6.68 | -5.42 |  |  |  |  |  |  |  |  |  |  |  |  |  |  | -7.62 | -7.75 |
| 1.49 | BX 37 |  |  |  |  |  |  |  |  |  |  |  |  | -4.98 | -1.55 |  |  |  |  |
| 1.41 | BX 36 |  |  |  |  |  |  |  |  |  |  |  |  | -5.29 | -0.34 |  |  |  |  |
| 1.17 | X 9 |  |  |  |  |  |  |  |  |  |  |  |  | -5 | -0.96 |  |  |  |  |
| 1.16 | BX 33 |  |  |  |  |  |  |  |  |  |  |  |  | -4.05 | 0.79 |  |  |  |  |
| 1.04 | BX 31 |  |  |  |  |  |  |  |  |  |  |  |  | -4.97 | 0.08 |  |  |  |  |
| 0.89 | X 7 |  |  |  |  |  |  |  |  |  |  |  |  | -4.38 | 0.15 |  |  |  |  |
| 0.88 | BX 29 |  |  |  |  |  |  |  |  |  |  |  |  | -3.36 | 1.02 |  |  |  |  |
| 0.85 | BX 28-2 |  |  |  |  |  |  |  |  |  |  |  |  | -4.47 | 0.46 |  |  |  |  |
| 0.84 | BX 28-1 |  |  |  |  |  |  |  |  |  |  |  |  | -4.51 | 0.21 |  |  |  |  |
| 0.81 | BX 26-2 |  |  |  |  |  |  |  |  |  |  |  |  | -4.25 | 0.88 |  |  |  |  |
| 0.80 | BX 26-1 |  |  |  |  |  |  |  |  |  |  |  |  | -5.39 | 1.28 |  |  |  |  |
| 0.78 | X 6 |  |  |  |  |  |  |  |  |  |  |  |  | -3.93 | 1.21 |  |  |  |  |
| 0.77 | BX 25 |  |  |  |  |  |  |  |  |  |  |  |  | -5.51 | 0.74 |  |  |  |  |

STab. 3. Isotopic values for bulk rock in sections VM1,2,3 and 4

| **SAMPLE** | **HEIGHT (m)** | δ **^13^C** | δ **^18^O_PDB_** | **UNIT** | **SECTION** |
| --- | --- | --- | --- | --- | --- |
| 220 | 2.2 | -5.91 | -4.81 | D | VM3 |
| 210 | 2.1 | -6.39 | -5.04 | D | VM3 |
| 200 | 2 | -6.28 | -4.67 | C2 | VM3 |
| 190 | 1.9 | -6.55 | -4.60 | C2 | VM3 |
| 180 | 1.8 | -6.39 | -4.40 | C2 | VM3 |
| 170 | 1.7 | -6.36 | -4.42 | C2 | VM3 |
| 160 | 1.6 | -5.52 | -4.43 | C2 | VM3 |
| 150 | 1.5 | -6.01 | -4.75 | C2 | VM3 |
| 140 | 1.4 | -6.47 | -4.83 | **C2** | VM3 |
| 130 | 1.3 | -7.14 | -4.79 | C1 | VM3 |
| 120 | 1.2 | -7.33 | -4.89 | C1 | VM3 |
| 110 | 1.1 | -8.23 | -4.72 | C1 | VM3 |
| 105 | 1.05 | -7.50 | -4.69 | C1 | VM3 |
| 100 | 1 | -7.25 | -4.99 | C1 | VM3 |
| 95 | 0.95 | -7.18 | -5.02 | C1 | VM3 |
| 90 | 0.9 | -7.74 | -5.10 | C1 | VM3 |
| 85 | 0.85 | -7.51 | -5.37 | C1 | VM3 |
| 80 | 0.8 | -7.15 | -5.87 | C1 | VM3 |
| 75 | 0.75 | -7.37 | -5.48 | C1 | VM3 |
| 70 | 0.7 | -7.00 | -5.85 | C1 | VM3 |
| 65 | 0.65 | -7.31 | -5.90 | C1 | VM3 |
| 60 | 0.6 | -7.40 | -5.76 | C1 | VM3 |
| 55 | 0.55 | -7.72 | -5.63 | C1 | VM3 |
| 50 | 0.5 | -7.05 | -5.77 | C1 | VM3 |
| 45 | 0.45 | -6.94 | -5.99 | C1 | VM3 |
| 40 | 0.4 | -7.38 | -5.93 | C1 | VM3 |
| 35 | 0.35 | -7.57 | -5.86 | C0 | VM3 |
| 30 | 0.3 | -7.06 | -5.76 | C0 | VM3 |
| 25 | 0.25 | -7.20 | -5.59 | C0 | VM3 |
| 20 | 0.2 | -7.04 | -5.54 | C0 | VM3 |
| 15 | 0.15 | -7.12 | -5.39 | C0 | VM3 |
| 10 | 0.1 | -7.01 | -5.55 | C0 | VM3 |
| 5 | 0.05 | -7.00 | -5.42 | C0 | VM3 |
| 0 | 0 | -7.01 | -5.42 | C0 | VM3 |
| VM17-070N | 2.12 | -6.11 | -5.18 | D | VM4 |
| VM17-080 | 2.02 | -7.09 | -5.35 | C2 | VM4 |
| VM17-090 | 1.92 | -7.17 | -5.19 | C2 | VM4 |
| VM17-100 | 1.82 | -7.05 | -5.07 | C2 | VM4 |
| VM17-110 | 1.72 | -7.11 | -5.01 | C2 | VM4 |
| VM17-120 | 1.62 | -6.7 | -5.46 | C2 | VM4 |
| VM17-130 | 1.52 | -6.88 | -5.85 | C2 | VM4 |
| VM17-140 | 1.42 | -7.36 | -5.65 | C2 | VM4 |
| VM17-145 | 1.37 | -7.67 | -6.21 | C1 | VM4 |
| VM17-150 | 1.32 | -7.63 | -5.52 | C1 | VM4 |
| VM17-155 | 1.27 | -7.6 | -5.53 | C1 | VM4 |
| VM17-160N | 1.22 | -7.33 | -5.91 | C1 | VM4 |
| VM17-165N | 1.17 | -6.98 | -6.1 | C1 | VM4 |
| VM17-170 | 1.12 | -7.19 | -6.22 | C1 | VM4 |
| VM-17-175 | 1.07 | -7.17 | -6.42 | C1 | VM4 |
| VM-17-180 | 1.02 | -7.01 | -6.11 | C1 | VM4 |
| VM17-185 | 0.97 | -7.5 | -6.56 | C1 | VM4 |
| VM17-190 | 0.92 | -6.99 | -6.2 | C1 | VM4 |
| VM17-195 | 0.87 | -6.96 | -6.16 | C1 | VM4 |
| VM17-200 | 0.82 | -6.8 | -6.03 | C1 | VM4 |
| VM17-205 | 0.77 | -6.91 | -6.14 | C1 | VM4 |
| VM17-210 | 0.72 | -7.14 | -6.17 | C1 | VM4 |
| VM17-215 | 0.67 | -6.93 | -5.77 | C1 | VM4 |
| VM17-220 | 0.62 | -7.31 | -6.15 | C1 | VM4 |
| VM17-225 | 0.57 | -7.13 | -5.87 | C1 | VM4 |
| VM17-230 | 0.52 | -6.89 | -5.84 | C1 | VM4 |
| VM17-235 | 0.47 | -6.6 | -5.67 | C0 | VM4 |
| VM05/2/05 | 5.3 | -6.14 | -6.04 | D | VM2 |
| ZG-05 TS | 3.6 | -5.83 | -6.10 | C2 | VM1 |
| ZG-05 TI | 3.55 | -5.95 | -6.32 | C2 | VM1 |
| ZG-05BS | 2.85 | -4.16 | -7.44 | C1 | VM1 |
| ZG-05 BI | 2.8 | -4.15 | -7.72 | C1 | VM1 |

**S2-SUMMARIZED STRATIGRAPHY AND RESULTS**

The general contribution of the previous data is summarized in an idealized section showing the stratigraphic units and intervals of the VM area:


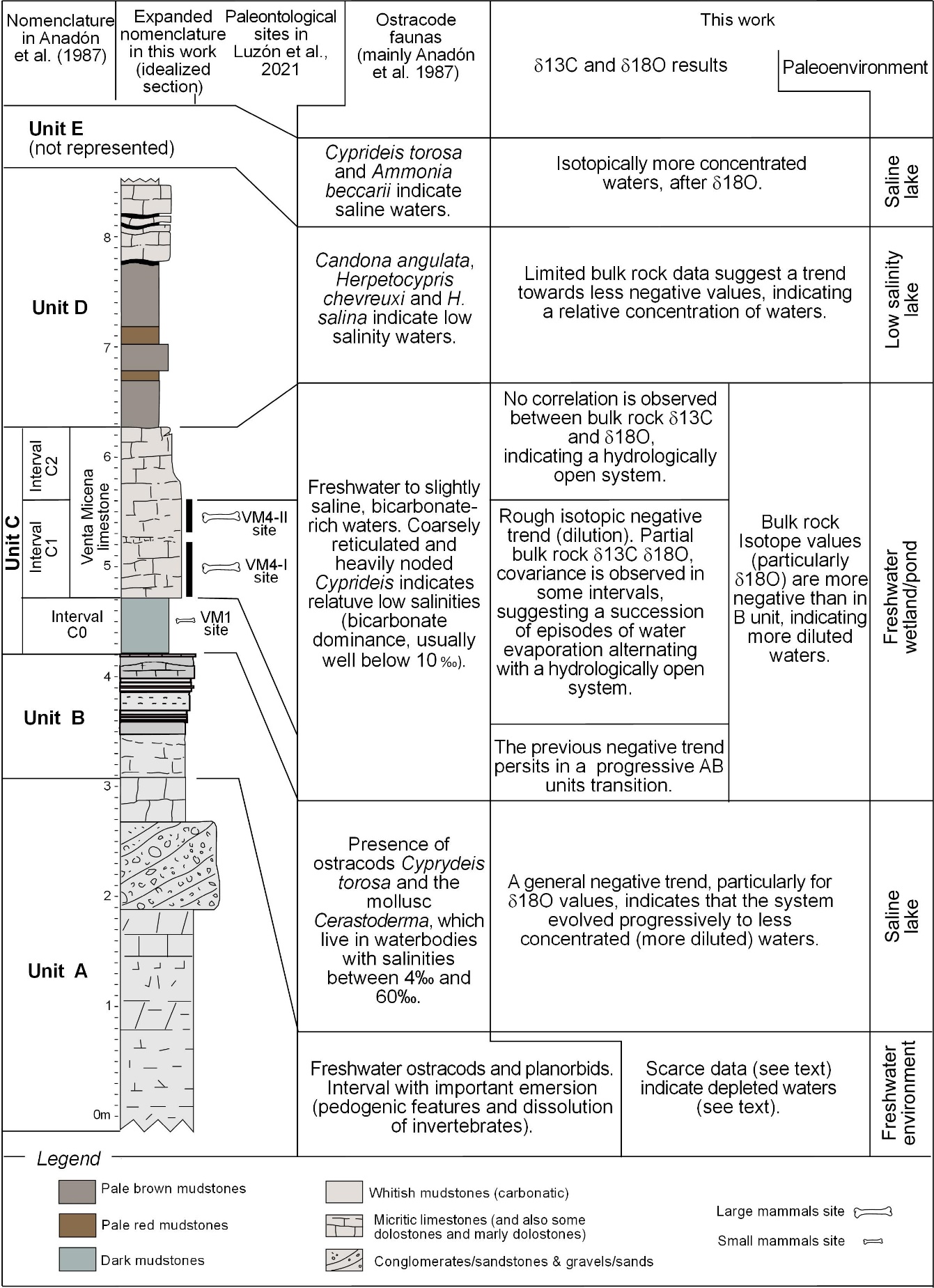


SFig. 2. Idealized Venta Micena section with the stratigraphic units by Anadón et al. (1987) together with the expanded stratigraphy by Luzón et al. (2021 and this study). A general summary of the results from this study is presented in the left columns.

**S3- CLUSTERING ANALYSIS OF STABLE ISOTOPES**

C1/2 clustering analysis


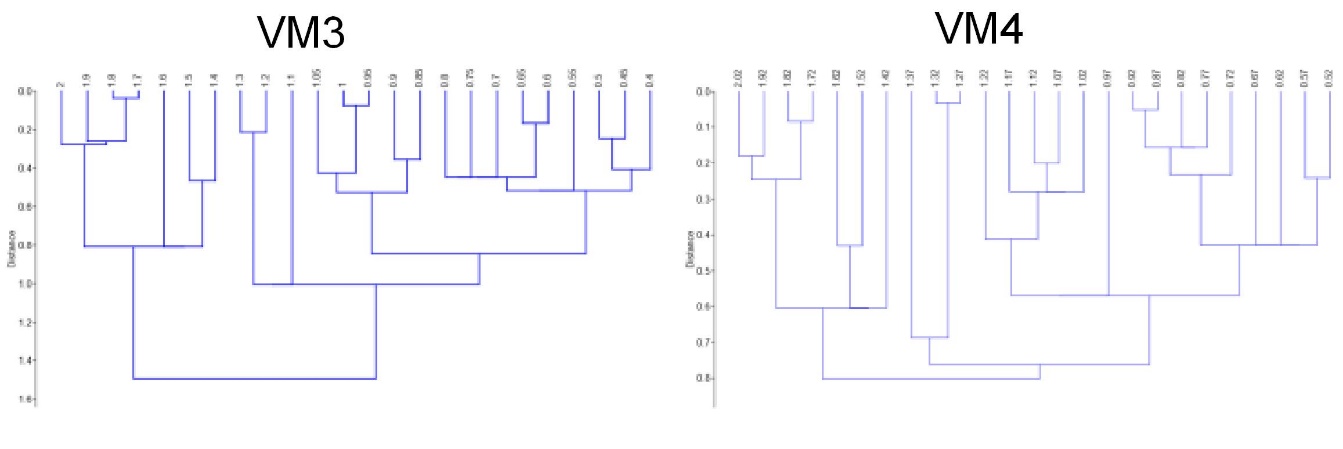


**SFig. 3.** Clustering analysis with stratigraphic constraints carried out in Past 3 software to better define the C1/C2 boundary on the basis of bulk rock O and C.

REFERENCES FOR SUPPLEMENTARY INFORMATION

Anadón, P., Oms, O., Riera, V. & Julià, R. The geochemistry of biogenic carbonates as a paleoenvironmental tool for the Lower Pleistocene Barranco León sequence (BL-5D, Baza Basin, Spain). Quat. Int. 389, 70–83 (2015).

Luzón, C. et al. Taphonomic and spatial analyses from the Early Pleistocene site of Venta Micena 4 (Orce, Guadix-Baza Basin, southern Spain). Sci. Reports 2021 111 11, 1–17 (2021).

Oms, O., Agustí, J., Gabàs, M. & Anadón, P. Lithostratigraphical correlation of micromammal sites and biostratigraphy of the Upper Pliocene to Lower Pleistocene in the Northeast Guadix‐Baza Basin (southern Spain). J. Quat. Sci. 15, 43–50 (2000).
